# Hip arthroscopy has good clinical outcomes in the treatment of osteoid osteoma of the acetabulum

**DOI:** 10.1186/s12891-021-04384-5

**Published:** 2021-05-28

**Authors:** Guanying Gao, Ruiqi Wu, Rongge Liu, Yingfang Ao, Jianquan Wang, Yan Xu

**Affiliations:** Institute of Sports Medicine, Beijing Key Laboratory of Sports Injuries, Peking University Third Hospital, 49 North Garden Road, Haidian District, 100191 Beijing, China

**Keywords:** Osteoid osteoma, Hip, Arthroscopy, Patient-reported outcomes

## Abstract

**Background:**

Osteoid osteoma (OO) of the acetabulum is a relatively rare disease. However, the the clinical outcomes of hip arthroscopy for treatment of OO of the acetabulum are still uncertain.

**Methods:**

We evaluated consecutive patients who were diagnosed with OO of the acetabulum and who underwent hip arthroscopy at our hospital between January 2013 and March 2020. All patients underwent a preoperative physical examination. Preoperative supine anteroposterior hip radiography, cross-table lateral radiographs, computed tomography (CT), and magnetic resonance imaging were performed in all patients. The alpha angle and lateral center-edge angle were measured before surgery. Supine anteroposterior hip radiography and CT were performed in all patients postoperatively. Preoperative patient-reported outcomes (PROs), including Visual Analog Scale (VAS), the International Hip Outcome Tool-12 (iHOT-12) and modified Harris Hip Score (mHHS), and PROs at final follow-up were evaluated.

**Results:**

A total of 6 patients (mean age, 18.7 years; age range, 6–31 years; 5 males and 1 females) were included in this study. The average follow-up period after surgery was 28.3 months (range, 6–90 months). Before surgery, the mean mHHS was 45.2 ± 10.5 (range, 33–56), the mean iHOT-12 was 33.3 ± 14.5 (range, 13–49), and mean VAS was 8.2 ± 1.0 (range, 7–9). At one month after surgery, mean mHHS was 78.7 ± 1.9 (range, 77–81), iHOT-12 was 71.0 ± 4.5 (range, 68–80), and mean VAS was 0. At the final post-operative follow-up, mean mHHS was 89.2 ± 2.1 (range, 86–91), iHOT-12 was 93.5 ± 5.0 (range, 88–98), and mean VAS was 0. All results, except VAS between one month after surgery and at final follow-up, demonstrated statistically significant improvement (*P* < 0.05). One patient underwent revision surgery.

**Conclusions:**

Hip arthroscopy has good clinical outcomes in the treatment of OO of the acetabulum. Further study on the mechanism of secondary femoroacetabular impingement (FAI) caused by OO of the acetabulum is needed. More cases of arthroscopic excision and longer follow-up are also needed to better prove the clinical outcomes of hip arthroscopy for OO of the acetabulum.

## Background

Osteoid osteoma (OO) is a small, benign, osseous neoplasm characterized by a nidus surrounded by reactive sclerotic bone with a size usually less than 20 mm [1–3]. Patients often present with local pain, worsening pain, pain at night, and pain that is relieved by non-steroidal anti-inflammatory drugs (NSAIDs) [4]. OO can be diagnosed using a combination of plain radiography, technetium-99 m bone scans, computed tomography (CT) scans, and magnetic resonance (MR) images [1]. Most cases of OO occur in the long bones of the lower extremities of patients in the second and third decades of life [5]. The femur and tibia are affected in > 50 % of cases; however, this type of tumor is rare in the pelvis and is difficult to diagnose [6–8]. OO of the acetabulum is even more rare. In recent literature, we found several case reports on treatment for OO of the acetabulum [1, 5, 9–23]. Minimally invasive percutaneous techniques, including CT-guided approaches and ablation using radiofrequency or lasers, as well as arthroscopic excision techniques for OO in acetabulum, have been described.

The purpose of this study was to evaluate the clinical outcomes of hip arthroscopy in the treatment of OO of the acetabulum. We hypothesized that hip arthroscopy could relieve symptoms, improve function and prevent recurrence in the treatment of OO of the acetabulum.

## Patients and methods

### Patients

We evaluated consecutive patients who were diagnosed with OO of the acetabulum and who underwent hip arthroscopy for treatment at our hospital between January 2013 and March 2020. The inclusion criteria were as follows: (1) patients who were diagnosed with OO of the acetabulum by clinical findings, plain radiography, CT, and MR images; and (2) underwent hip arthroscopy with (3) postoperative pathological confirmation of OO. Patients who could not complete the clinical follow-up were excluded from the study. All participants signed informed consent. The study was approved by the Ethics Committee of the Third Hospital of Peking University. All methods were performed in accordance with the guidelines and regulations of the Ethics Committee of the Third Hospital of Peking University.

### Physical examination and radiographic assessment

**Fig. 1:**
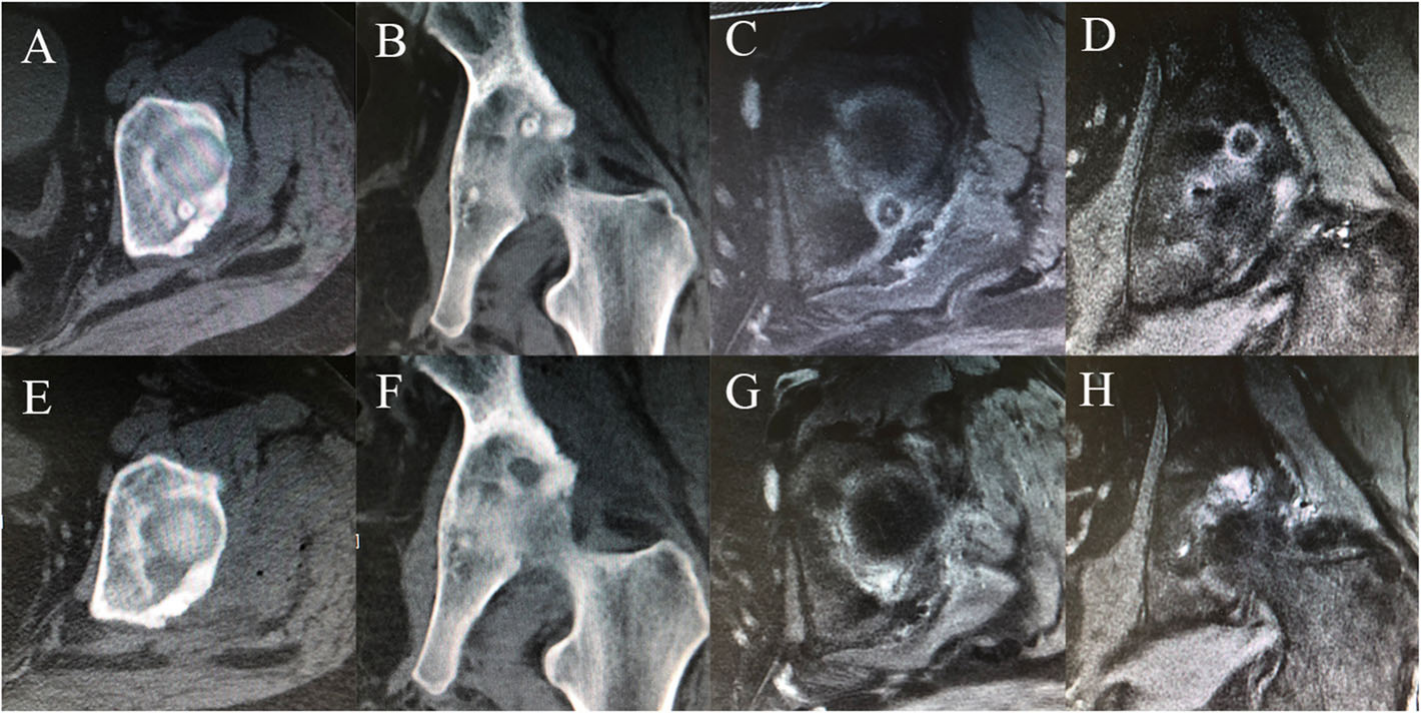
Preoperative and postoperative CT and MRI of one patient diagnosed with OO of the acetabulum in zone 4. **A-D.** Preoperative axial CT, coronal CT, axial MRI and coronal MRI showed the location of OO. **E-H**. Postoperative axial CT, coronal CT, axial MRI and coronal MRI showed the excision of OO

**Fig. 2:**
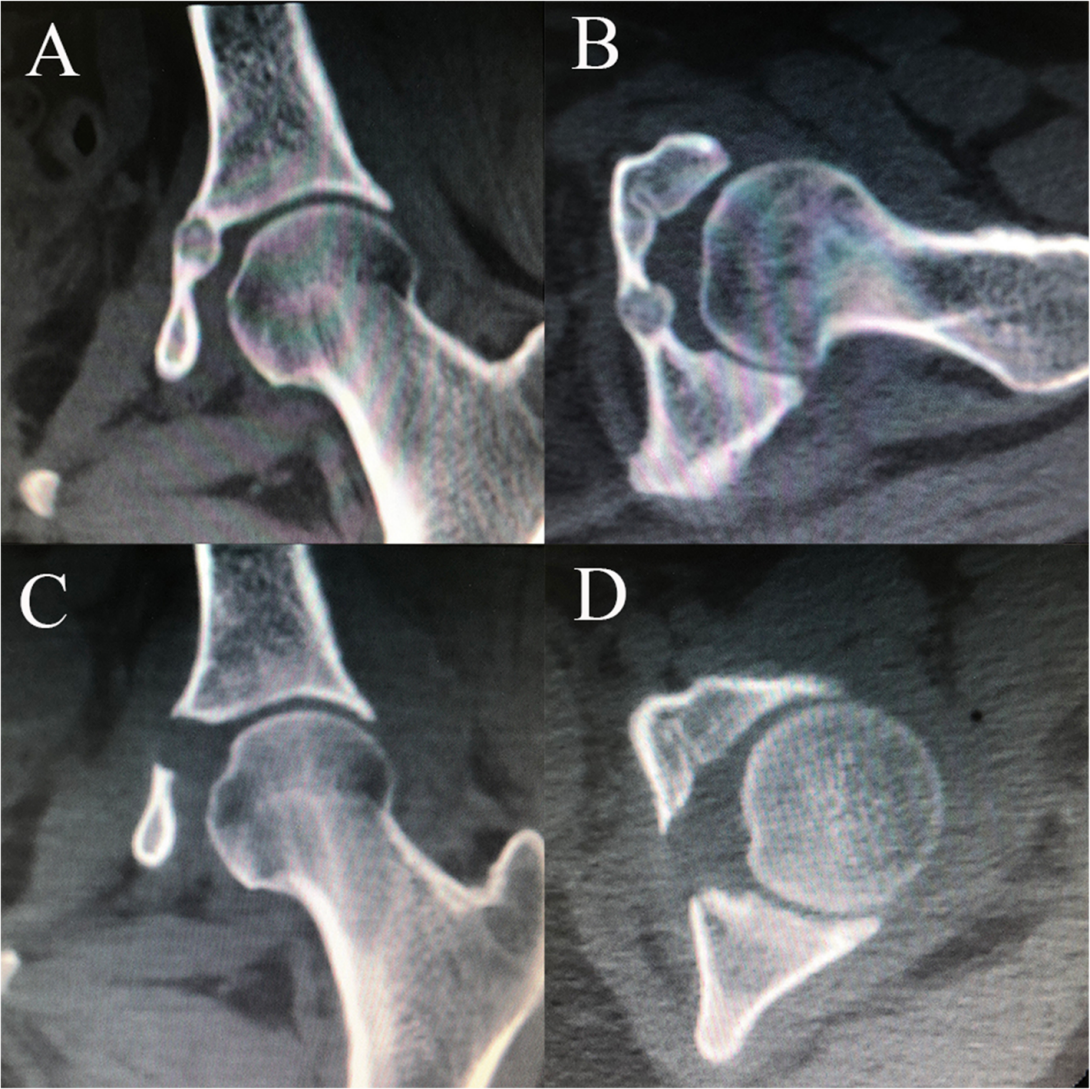
Preoperative and postoperative CT of one patient diagnosed with OO of the acetabulum in zone 6. **A, C**. Preoperative and postoperative coronal CT. **B, D**. Preoperative and postoperative axial CT

All patients underwent a thorough and systematic physical examination, including specific tests previously described for diagnosing hip diseases [24]. Flexion, adduction, and internal rotation (FADIR) or flexion, abduction, and external rotation (FABER) tests were considered positive if hip or groin pain was elicited when the hip was placed at 90° of flexion, followed by adduction and internal rotation, or flexion, abduction, and external rotation [25]. Supine anteroposterior hip radiography, cross-table lateral radiography, CT, and MR images were performed on all patients preoperatively (Figs. 1 and 2). Cross-table lateral radiography and CT were performed on all patients postoperatively. The preoperative alpha angle and lateral center-edge angle (LCEA) were measured as described previously [26, 27].

### Surgical technique and postoperative rehabilitation protocol

One surgeon with more than 10 years of experience performed standard hip joint arthroscopy on all patients. All surgeries were performed using a standard supine approach as described by Gao et al. [28]. The operation was performed under spinal anesthesia. In brief, a detailed inspection of the central compartment was performed to assess the acetabular rim, acetabular labrum, articular cartilage, and ligamentum teres. Labral repair or labral debridement was performed according to the nature of injury. If a cam bump in the head-neck junction or acetabular overcoverage was identified, femoral osteoplasty or acetabuloplasty was performed. The degree of cartilage damages was assessed according to the Outerbridge classification system [29]. To identify the location of OO, we increased the force of traction for better exposure and vision. After OO was identified, a fine guide wire was placed to mark the nidus. Then, the C-arm was used to confirm that the identified nidus matched what was seen on preoperative radiographic images. The tumor and surrounding sclerotic bone tissue were then removed using a narrow bone knife and an abrasive drill until normal cancellous bone was reached. Radiofrequency was used to stabilize the cartilage around the tumor. Cartilage deficiency following excision was not addressed because the resected area was relatively small. The posterior area of the hip is usually difficult to access in arthroscopy. A 70 degrees arthroscope and flexible instruments could help remove lesions. The location of OO was recorded according to a geographic zone method described by Ilizaliturri et al. [30]. The acetabulum was divided into six zones as follows: the anterior inferior (zone 1), the anterior superior (zone 2), the middle superior (zone 3), the posterior superior (zone 4), the posterior inferior (zone 5) and the middle inferior (zone 6; cotyloid fossa). After the treatment of the central compartment, the lower extremities were relaxed, and the arthroscope was inserted into the peripheral compartment. Capsular closure was routinely performed at the end of surgery.

A routine postoperative rehabilitation protocol with modifications was used, as described by Gao et al. [28]. In brief, ankle pump, quadriceps strengthening and other isometric exercises were initiated 1 days after surgery. Hip passive range of motion (ROM) exercises were started as tolerated three days after surgery. Partial weight bearing with crutches was started 3 days after surgery, and passive ROM exercises and active ROM exercises were performed as tolerated after 2 postoperative weeks. Patients were encouraged to advance to full weight bearing by 4 postoperative weeks, and we aimed to restore symmetrical hip ROM 4 weeks after surgery. Patients were permitted to begin jogging and advance to running 3 months after surgery.

### Clinical evaluation

Preoperative patient-reported outcomes (PROs) and PROs one month after surgery and at final follow-up were obtained, including visual analog scale (VAS) for pain, the International Hip Outcome Tool-12 (iHOT-12) and modified Harris Hip Score (mHHS). We used thresholds defined for PROs commonly used in the hip preservation literature. For the mHHS, the minimal clinically important difference (MCID) was defined as 8 by Kemp et al. [31], and the patient acceptable symptom state (PASS) score was defined as 74 by Chahal et al. [32]. For iHOT-12, the MCID was determined as 13 by Martin et al. [33] and the PASS was determined to be 63 by Nwachukwu et al. [34]. Complications and revision hip arthroscopy were recorded.

### Statistics

A two-tailed paired t-test was used to evaluate significance between preoperative and postoperative PROs. *P* values of < 0.05 were considered statistically significant. All statistical analyses were performed with SPSS Statistics, version 22 (IBM).

## Results

As shown in Table 1, a total of 6 patients (mean age, 18.7 years; age range, 6–31 years; 5 males and 1 females) were included in this study. There were five cases of left-sided OO and one case of right-side OO. The mean body mass index (BMI) was 20.6 (range, 12.4–33.2). The mean duration of pain before surgery in our hospital was 17.5 months (range, 6–36 months). Five patients (83.3 %) experienced worsening of pain at night, while one patient experienced the same degree of pain during the day compared with at night. Five patients (83.3 %) achieved pain relief after taking NSAIDs, while one patient did not take NSAIDs. Three patients (50 %) underwent previous surgery at another hospital and underwent revision surgery at our hospital. OO of the acetabulum in 2 of these 3 patients was misdiagnosed, and these 2 patients only underwent femoral osteoplasty and labral repair as their primary surgery. One patient had a correct diagnose of OO of the acetabulum and underwent radiofrequency ablation guided by CT at another hospital. However, none of these three patients achieved pain relief after primary surgery; thus, they attended our hospital. The FADIR test, as evaluated by the treating physician, was positive in 4 patients (66.7 %), while the FABER test was positive in 5 patients (83.3 %). In addition, 3 patients experienced tenderness in the groin area, 2 patients experienced tenderness in the posterior hip, 2 patients experienced tenderness over the greater trochanter, 1 experienced tenderness in the sacroiliac joint and 1 experienced tenderness in the posterior superior iliac spine. Mean preoperative alpha angle and LCEA were 62.4 ± 12.9° (range, 50.6–79.6) and 33.4 ± 4.5° (range, 28.1–40.6), respectively.

**Table 1:** Demography of patients (*n* = 6)

Parameter	Data
Age, y, mean (range)	18.7 (6–31)
Sex	
Male	5 (83.3 %)
Female	1 (16.7 %)
BMI, kg/m^2^, mean (range)	20.6 (12.4–33.2)
FADIR test	
Positive	4 (66.7 %)
Negative	2 (33.3 %)
FABER test	
Positive	5 (83.3 %)
Negative	1 (16.7 %)
Duration of pain (range)	17.5 (6–36)
Alpha angle (range)	62.4 (50.6–79.6)
LCEA (range)	33.4 (28.1–40.6)

NOTE. Unless otherwise specified, data are numbers of patients, with percentages in parentheses

**Fig. 3:**
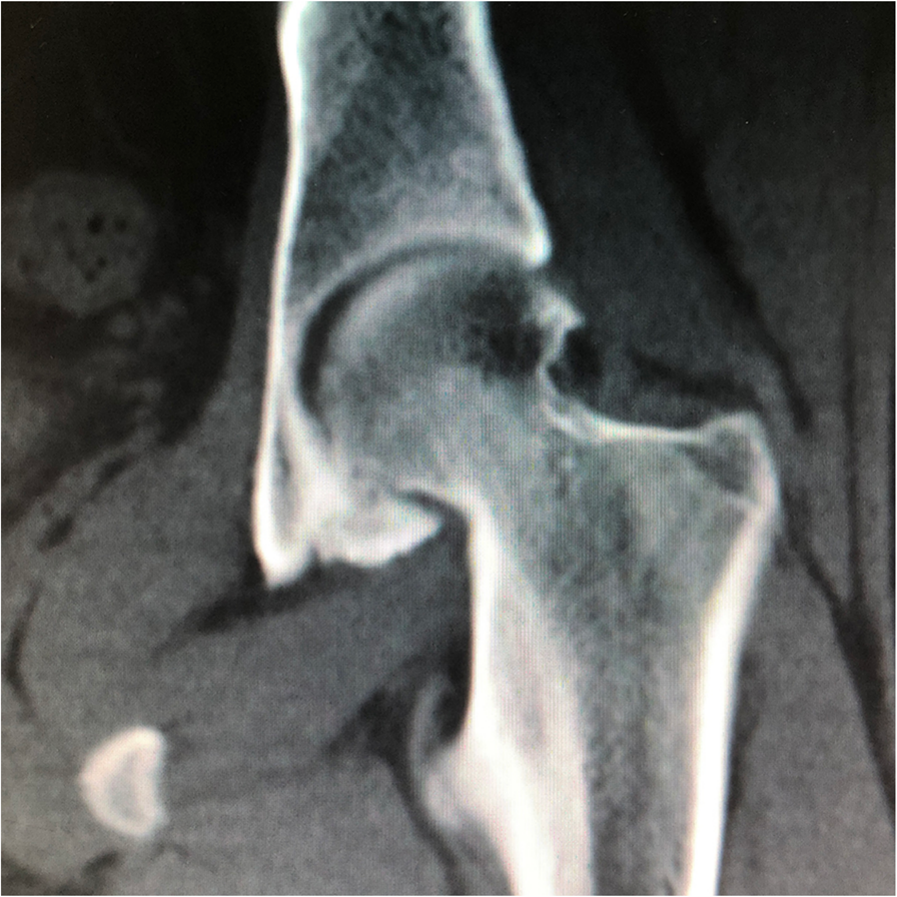
Coronal CT showing OO and a periosteal reaction in the joint surface of the acetabulum

The arthroscopic and radiographic diagnoses of patients were shown in Table 2. Among these 6 patients, 4 patients (66.7 %) were diagnosed with combined femoroacetabular impingement (FAI), one patient was diagnosed with Tonnis grade 1 osteoarthritis (OA) by anteroposterior hip radiography, and two patients were found to have a periosteal reaction in the joint surface of the acetabulum (Fig. 3). All 6 patients underwent arthroscopic excision of OO, 4 patients underwent femoral osteoplasty, 1 patients underwent acetabuloplasty, and 2 patients underwent labral repair. OO of the acetabulum was located in zone 5 in 4 patients (66.7 %), in zone 4 in 1 patient (16.7 %), and in zone 6 in 1 patient (16.7 %). Two patients (33.3 %) had Outerbridge I or II femoral cartilage damages, 1 (16.7 %) who had Outerbridge IV femoral cartilage damages, 1 (16.7 %) who had Outerbridge II acetabular cartilage damages, and 3 patients (50 %) who had Outerbridge III acetabular cartilage damages.

**Table 2:** Diagnosis and arthroscopic findings

	Data
Diagnosis	
OO of the acetabulum	6 (100 %)
Cam impingement	4 (66.7 %)
Pincer impingement	1 (16.7 %)
Acetabular labral tear	3 (50 %)
Osteoarthritis	1 (16.7 %)
Location of OO	
Zone 4	1 (16.7 %)
Zone 5	4 (66.7 %)
Zone 6	1 (16.7 %)
Degree of femoral cartilage damages	
0	3 (50 %)
I	1 (16.7 %)
II	1 (16.7 %)
III	0
IV	1 (16.7 %)
Degree of acetabular cartilage damages	
0	2 (33.3 %)
I	0
II	1 (16.7 %)
III	3 (50 %)
IV	0

NOTE. Unless otherwise specified, data are presented as numbers of patients, with percentages in parentheses

The average follow-up period after surgery was 28.3 months (range, 6–90 months). Pain disappeared immediately after surgery in all patients. As shown in Table 3, before surgery, the mean mHHS was 45.2 ± 10.5 (range, 33–56), the mean iHOT-12 was 33.3 ± 14.5 (range, 13–49), and mean VAS was 8.2 ± 1.0 (range, 7–9). At one month after surgery, the mean mHHS was 78.7 ± 1.9 (range, 77–81), the mean iHOT-12 was 71.0 ± 4.5 (range, 68–80), and mean VAS was 0. At the final post-operative follow-up, the mean mHHS was 89.2 ± 2.1 (range, 86–91), the mean iHOT-12 was 93.5 ± 5.0 (range, 88–98), and mean VAS was 0. All results, except VAS between one month after surgery and at final follow-up, demonstrated statistically significant improvement (*P* < 0.05). All patients surpassed the 2 and achieved the PASS for mHHS and iHOT-12 one month after surgery and at final follow-up. No complications were recorded during the study period. One patient still had pain after surgery and underwent revision arthroscopy because of excision at the wrong position. This patient achieved a complete pain relief after revision surgery.

**Table 3:** Patient-reported outcomes

PROs	Before surgery	One month after surgery	Final follow-up
mHHS	45.2 ± 10.5	78.7 ± 1.9	89.2 ± 2.1
iHOT-12	33.3 ± 14.5	71.0 ± 4.5	93.5 ± 5.0
VAS	8.2 ± 1.0	0	0

NOTE. Values are presented as mean ± standard deviation

## Discussion

In this study, we found that hip arthroscopy has good clinical outcomes in the treatment of OO of the acetabulum. Pain disappeared immediately after surgery in all patients. The mHHS and iHOT-12 improved significantly one month after surgery and at final follow-up. All patients surpassed the MCID and achieved the PASS for mHHS and iHOT-12 one month after surgery and at final follow-up. VAS improved significantly one month after surgery. There was no significant difference in VAS one month after surgery compared with at final follow-up. The pain disappeared immediately after surgery in all patients.

OO of the acetabulum can be difficult to diagnose. A delay in diagnosis may lead to muscle atrophy, tenderness, localized swelling, possible contractures, articular damage and early OA [6]. Previous studies have proven the effectiveness of NSAIDs for the treatment of OO, and cases of spontaneous healing of OO treated with NSAIDs have been reported [4, 35]. In this study, patients with OO of the acetabulum can also achieved pain relief using of NSAIDs. In recent researches, percutaneous resection guided by CT scan, radiofrequency ablation, arthroscopy-assisted radiofrequency ablation, and arthroscopic excision for the treatment of OO of the acetabulum have been reported [1, 5, 9–13, 15, 17–19, 22]. With CT-guided ablation, destruction of the articular cartilage around the lesion is unavoidable, and a specimen for pathologic examination may not be obtainable because of thermal damage [8, 19, 36]. Mortensen et al. reported a 15-year-old male who was treated with radiofrequency ablation for OO of the acetabulum after failure of primary hip arthroscopy for misdiagnosed FAI. The patient reported high satisfaction and minimal pain at 3 years of follow-up, which proved the feasibility of CT-guided percutaneous radiofrequency ablation. The advantages of arthroscopy are less surgical damage, accurate targeting and excision of the lesion, and treatment of any resultant cartilage damage [12]. Synovectomy can be also be performed during arthroscopic lesion removal, which may prevent cartilage damage, speed up healing, and immediately relieve pain [4]. Dai et al. [20] retrospectively evaluated 25 patients who underwent arthroscopic excision for hip OO, including 22 cases on femoral side and 3 in the acetabulum. They reported great improvement in mHHS and iHOT-12. However, the authors did not analyze OO of the acetabulum separately and the clinical outcomes of arthroscopic excision of OO of the acetabulum were thus still unclear. Eberhardt et al. [37] also reported 3 cases of OO of the acetabulum treated by hip arthroscopy. We found descriptions of several cases using arthroscopic excision and one case using arthroscopy-assisted radiofrequency ablation for the treatment of the OO of the acetabulum [1, 5, 9, 10, 12, 17, 20, 21, 23, 37]; however, data on patients treated using by hip arthroscopy alone were scare.

In the present study, OO of the acetabulum in 2 of 6 patients (33.3 %) was misdiagnosed at another hospital, and the 2 patients only underwent femoral osteoplasty and labral repair only as their primary surgery. OO of the acetabulum is easy to be misdiagnosed and this feature has been previously described [6, 12, 38]. Two patients in our study underwent revision surgery after CT-guided radiofrequency ablation and arthroscopic excision. Sometimes, it is indeed difficult to locate the lesion under arthroscopy. In some patients, cartilage changes could be observed on the surface of the lesion, which helped to identify the lesion. However, sometimes no abnormality is observed in cartilage.

It should be noticed that OO of the acetabulum in 4 of 6 patients (66.7 %) was located in zone 5 in this study. The other two cases of OO were located in zones 4 and 6. In the existing studies on arthroscopic treatment of OO of the acetabulum described above, two cases were located in the posterior area [9, 10], one was located in the posteroinferior area [13], one was located at the bottom of the acetabulum [1], one was located in the superior portion of the acetabulum [12], and one (a 10-year-old boy) was located under the triradiate cartilage [17]. Thus, we concluded that, of all cases of acetabular OO, the frequency of OO in the posterior acetabulum is high. In our clinical work, we need to focus on this area. Although we used a 70 degrees arthroscope and flexible instruments to perform arthroscopic excision, it is usually difficult and time-consuming to get access to the posterior area of acetabulum, especially to zone 5. Excision of OO of the acetabulum in the posterior area of the hip requires suitable equipment, patience, and experience.

In this study, 4 of 6 patients (66.7 %) had concomitant FAI. We thought that FAI in these patients was secondary to OO. Three patients (50 %) had concomitant labral tear caused by secondary FAI. We thought that OO causes a repeated inflammatory reaction and bone hyperplasia, which could lead to secondary FAI. Bone hyperplasia of the acetabular fossa and relative lateral movement of the femoral head may cause secondary FAI. Further study on secondary FAI is needed in the future.

In addition, one patient in this study had sclerosis of the acetabulum and slight narrowing of the joint space and was diagnosed with Tonnis grade 1 OA. Norman et al. [39] evaluated 30 patients with intraarticular OO of the hip and found that OA developed in 50 % of those patients. OO of the hip could stimulate an early-onset OA. Repeated inflammatory reactions that damaged cartilage may lead to OA caused by OO of the hip. OO of the acetabulum on the joint surface may lead to more direct and severe irritation. Moreover, two patients had a periosteal reaction in the joint surface of the acetabulum, which could be a diagnostic feature of OO of the acetabulum.

### Limitations

This study has some potential limitations that should be noted. Firstly, this study only included a small sample size due to the rarity of OO of the acetabulum. Secondly, two patients were only followed up for a relatively short period of time. We found that pain disappeared immediately after surgery in all patients, so we thought that the therapeutic effect of arthroscopic excision of OO of the acetabulum could be achieved in a short period of time.

## Conclusions

Hip arthroscopy has good clinical outcomes in the treatment of OO of the acetabulum. Further study on the mechanism of secondary FAI caused by OO of the acetabulum is needed. Furthermore, more cases of arthroscopic excision and longer follow-up are also needed to better prove the clinical outcomes of hip arthroscopy for OO of the acetabulum.

## Data Availability

All relevant data supporting the conclusions are included within the article and tables. The datasets used and/or analysed during the current study available from the corresponding author on reasonable request.
